# If You Don’t Eat Meat… You’ll Die. A Mixed-Method Survey of Health-Professionals’ Beliefs

**DOI:** 10.3390/nu11123028

**Published:** 2019-12-11

**Authors:** Patrick McHugh, Morgen Smith, Nicholas Wright, Sarah Bush, Sue Pullon

**Affiliations:** 1 Te Hauora O Turanganui-A-Kiwa (Turanga Health), 4010 Gisborne, New Zealand; 2 Plant-Based New Zealand Health Charity, 4010 Gisborne, New Zealand; Morgensm@gmail.com (M.S.); nicholas.samuel.wright@gmail.com (N.W.); 3 Bioethics Centre (Te Pokapū Matatika Koiora), University of Otago, 9054 Dunedin, New Zealand; s.bush@hotmail.co.nz; 4 Department of Primary Health Care and General Practice (Te Whare Wānanga o Otāgo ki Te Whanga-Nui-a-Tara), University of Otago, 6242 Wellington, New Zealand; sue.pullon@otago.ac.nz

**Keywords:** diet, plant-based diet, vegan diet, vegetarian diet, low-fat diet, whole food plant-based diet, nutrition, education, health workforce

## Abstract

Despite an ever-increasing burden of non-communicable diseases and overwhelming evidence that good nutrition improves outcomes it is difficult to know whether this evidence is reaching the general population. The purpose of this study was to investigate whether health professionals in Tairāwhiti have sufficient nutrition education for their roles in health education and promotion and whether nutrition beliefs held by health professionals were consistent with current literature. A particular interest was to enlist views on the harms, benefits, and possible barriers to following plant-based diets. A mixed-methods study involving health professionals completing a questionnaire and a subsequent focus group to collect data was used. Survey data were analysed using spreadsheet software, and thematic content analysis of focus group data was undertaken. Participants provided nutrition advice 2.4 times per day. Almost half of practitioners considered their nutrition knowledge to be inadequate, and most made poor use of references for provision of information. Plant-based diets were generally viewed as beneficial to health, improve quality of life, be filling, but were perceived as not as easy to follow. This study is in keeping with previous research that the health workforce would benefit from more formalised nutrition education and competencies to address common chronic disease.

## 1. Introduction

Poor diet is the leading cause of disease and death globally; killing 11 million people a year [1]. In combination, lifestyle-related chronic disease kills 41 million people a year–71% of all deaths [2]. As is similar to many developing countries, New Zealand spends 77% (2.9 of the 3.8 billion USD per year) of the total healthcare costs on non-communicable disease treatment, many of which relate to excess calories and poor diet, alongside other lifestyle-related chronic disease risk-factors [3]. The expectation is that these costs will continue to rise as the burden of chronic illness such as cardiovascular disease cancer; diabetes; and obesity, continues to worsen. With the strong links between dietary choices and chronic disease, e.g., cardiovascular disease [4], healthcare services need to be responsive to their consumers and this would ideally include a primary healthcare workforce with well-developed competency for evidence-based counselling of nutrition for lifestyle diseases.

Previous research on dietary education in developed countries has revealed that education is lacking, however. For example, a recent Lancet systematic review of nutrition in medical students’ education concluded: “medical students are not supported to provide high-quality, effective nutrition care” and suggested revisions to curricula including compulsory education and standardised nutritional competencies [5]. United States based research revealed a “compelling need to markedly improve nutrition education” and very similar recommendations [6]. In New Zealand, research on General Practitioners and medical students showed “moderate confidence towards incorporating nutrition care into practice”. The sampling included those GPs attending a nutrition workshop, potentially over-estimating baseline knowledge, but it also concluded that more information was needed [7].

In New Zealand, medical students receive in the order of 20 h of nutrition education [8] during their training. American medical schools expect 25 h of nutrition training although the national delivered average is 19 h, with graduates generally feeling dissatisfied with nutrition education [9]. Nutrition education is regarded as a core competency for nursing [6], and the majority of community pharmacists also feel inadequately prepared with their nutrition education [10,11].

This study investigated nutrition knowledge, attitudes to nutrition, and perceptions about nutritional education of a small group of various healthcare practitioners in the Tairāwhiti (Gisborne) region of New Zealand. The BROAD study [12], a randomised controlled trial of a whole food plant-based (WFPB) diet programme was conducted in Gisborne, New Zealand, prior to this study. A WFPB diet strictly excludes animal products, and emphasises whole foods, and does not restrict quantities of food, whereas a “plant-based” diet provides the majority of calories from plant food. The impetus for the study came after a patient participating in a lifestyle study reported she was informed by a healthcare professional that “if you don’t eat meat, you’ll die!”, with an ensuing discussion around experiences of patients and health professionals regarding nutritional “myths”. The purpose of this study was therefore to investigate whether health professionals in Tairāwhiti had sufficient nutrition education for their roles in health education and promotion and whether nutrition beliefs held by health professionals were consistent with the current literature. A particular interest of the study was to acquire views from local health practitioners on the harms or benefits of a plant-based diet and explore “nutritional myths”. Previous research on WFPB diets has shown numerous benefits, not limited to: reversal of ischaemic heart disease [13]; greater weight loss than other dietary approaches [12]; improvements with diabetes [14]; and that plant-based diets are more environmentally sustainable than other approaches [15].

## 2. Materials and Methods

A mixed-method study involving a health professional survey and focus group was used to collect data to explore healthcare professionals’ views and beliefs about nutrition with a specific emphasis on plant-based diets. All research relating to this work was done in the Gisborne region. Data were collected by two methods:

1. A paper-based survey made available to health professionals in their workplaces. We left survey forms at: three general practices; three pharmacies; a long-term conditions unit at the local hospital; a staff break area at the hospital which was accessible by doctors, pharmacists, and nurses; and also an osteopathic medicine clinic. The paper-based survey forms were only accessible to health professional staff, and were filled in at leisure. Total numbers of staff that could have had access to the invitation forms in the various workplaces would have been between the order of 80–120, although we have no way of knowing how many would have in fact seen the invitation to participate. We also spoke to the individual staff present and added signage within the break areas asking that staff please participate.
2. A focus group of individuals that completed the survey and consented to participate in a focus group.

### 2.1. How Surveys Were Used

The surveys and focus group were performed between November 2017 and January 2018. For the survey, health professionals working in the community, likely to be asked for or to be discussing nutritional advice, were invited to participate in the survey. Local general practices, community clinics, pharmacies, and a nurse-led long-term condition clinic were approached for permission to distribute surveys. Within general practices, the practice manager, or an equivalent person was approached for permission to distribute the surveys and, where appropriate, asked to inform staff about the survey. Surveys were deposited at the survey locations in an area accessed by staff, e.g., staff tearoom, with survey instructions and a deposit box for returning completed anonymous surveys. The completed surveys were collected and removed at least twice a week. Various health professions were approached to provide a cross-section of the health care population.

### 2.2. Development of the Survey

The questionnaire and information sheet used for the survey was designed de novo after an on-line search was unsuccessful in locating a fit-for-purpose already validated questionnaire. The questionnaire was piloted with several healthcare practitioners not participating in the trial and further refined. While the survey data were anonymously collected, general demographical data was also collected and several questions allowed space for freehand comments. The final page of the questionnaire included an easily detachable consent form for any participants that were willing to be approached to be part of a focus group, to be placed in the survey deposit box separately to the questionnaire. The questionnaires and focus group consent forms were not analysed together to preserve the anonymity of those answering the surveys.

### 2.3. Focus Group

Subsequently, one focus group session was completed with four consenting health practitioners. The focus group was held with a health researcher facilitating the hour-long session whilst a second researcher observed and took notes. The participants were offered a $40 fuel voucher for their time. The session was recorded and stored on a password-protected computer that only the research team could access. Survey data was collated and analysed using spreadsheets and thematic content analysis from the focus group was undertaken. 

Ethics approval was granted by the University of Otago Human Ethics Committee, Category B (D17/383).

## 3. Results

The survey group of health professionals was made up of 73% New Zealand European, 12% Maori, 7% Chinese and other ethnicities (12%) (several participants gave more than one ethnicity). There was a reasonable cross-section of healthcare professionals (Table 1), with half of the participants being doctors, many had significant postgraduate experience, and participants estimating that on average they gave out nutritional advice 2.4 times per day. Local dieticians were invited but collectively declined to participate.

A total of 41 survey responses were received and one focus group was held. Participants recalled a wide range of estimated hours of nutrition education, both prior to (0 to 550 h, *n =* 22) and following (0 to 200 h, *n =* 19) graduation although survey participants found this estimate challenging (often having to remember back many years) with around half of the cohort not fully completing this section. Using available data, participants reported the following nutritional training: prior to graduation 55% had 10 or fewer hours (median 7.5); post-graduation 48% had 10 or fewer hours (median 20).

### 3.1. Perceived Adequacy of Nutrition Education

Of particular interest, 43% of participants reported dissatisfaction with the amount of nutritional training for their current role (Table 2). Focus group and survey comment responses reported significant learning was self-directed with wide-ranging examples of self-directed learning given during the focus group:

“A two-day nutrition course run by Heart Foundation–for anyone, not just health professionals”

*(Doctor)*

“Post-grad paper with one dietitian lecture”

*(Pharmacist)*

“Post-grad cancer nursing paper, … with 1.5 h dietitian lecture”

*(Nurse)*

“Chose to sit in with dietician clinic for own patients”

*(Nurse)*

### 3.2. Evidence and Guidelines Referenced by Study Participants

Responses were many and varied when participants were asked to provide guidelines or evidence in reference to what they perceived to be an “ideal diet”. Of the 41 responses, 10 (24%) gave no answer, 4 (10%) were unsure, 4 (10%) reported “own experience”, 11 (27%) referred to unspecified studies and 10 (24%) gave specifically named references. Two participants specifically noted within their responses that no “one” diet was ideal:

*“Everyone is different” *

*(Pharmacist and Doctor–in separate survey responses)*

*“Increasing evidence in genetic studies that there is a genetic basis to people’s response to different diets” *

*(Doctor–survey response)*

The Heart Foundation (cited by 12% of survey respondents) [16] and Diabetes Guidelines (cited by 10%) [17] were the most commonly named specific sources/references for information with less specific sources including “Mediterranean Diet” and “The Blue Zones” (a study of the longest-lived populations globally [18]):

*“Eat food, not too much, mainly plants”.*

*(Pharmacist – survey response – quoting Michael Pollan)* [19]

### 3.3. Perceptions of the Healthfulness of Various Dietary Approaches

There was a consensus amongst participants that diets high in processed meats were unhealthy and that a high intake of vegetables was healthy. One or more eggs per day were perceived as slightly healthy and three or more servings of dairy or milk per day more likely to be unhealthy than healthy (See Table 3). However, dairy products, and in particular milk, were considered to be the best sources of calcium, though vegetables were also considered an important source. Nearly all (94%) of the respondents thought that a high calcium diet was required for bone strength and to prevent osteoporosis. Meat was considered the best source of both protein and iron by 73% and 87% of respondents, respectively, though vegetables and legumes were also common responses. Survey respondents thought protein was considered important for muscle maintenance and function, while iron was required for red blood cell function.

### 3.4. Dietary Requirements in Various Disease States

Study participants were asked about chronic illness generically, then specific disease states. Study participants agreed that in the context of chronic illness every attempt should be made to eat a healthier diet (80% of respondents agreeing or strongly agreeing) (See Table 4). Attitudes regarding attempts to eat healthier were maintained with “very advanced” kidney disease (61% of respondents agreeing or strongly agreeing) and very advanced heart disease (56% of respondents agreeing or strongly agreeing) but not with very advanced cancer where only 17% of respondents agreed or strongly agreed. One participant commented regarding advanced cancer; “It’s chippies and ice cream time” (Doctor–survey response).

The majority of participants believed that atherosclerosis could be reversed using dietary means (67% True, 31% Not sure, 3% False, *n =* 39). However, focus group members thought that improving nutrition for patients who had suffered a heart attack was to be considered secondary to starting medications. Vegetarian, vegan and plant-based diets were generally thought to be beneficial (See Table 5) and a high-meat diet harmful. Participants believed that to follow a plant-based diet (See Table 6) would, in the main, be complicated, but also be filling and lead to an improvement in perceived quality of life.

Common barriers suggested to starting a plant-based diet included cost, access to foods, difficulty in preparing meals (particularly when managing family meals), and that; “It would be hard to break the habit of eating meat, especially when influenced by New Zealand culture around eating meat” (Doctor–focus group).

## 4. Discussions

This study investigated the nutritional knowledge of non-dietitian healthcare practitioners in Tairāwhiti. The surveyed healthcare practitioners were split in their perceptions of the adequacy of nutrition training for their current role; 43% perceived a paucity of nutrition education. Participants reported a wide range of exposure to nutrition education both during (median 7.5 h; range 0 to 550) and post-training (median 20 h; range 0 to 200). From the survey and focus group, it appears much of the learning was self-directed and done out of interest. Professionals were providing nutrition advice to patients, on average, 2.4 times per day. Awareness of and reported application of evidence-based nutrition guidelines were low with 51% of participants reporting unspecified studies and references. “Heart Foundation Guidelines” and “Diabetes Guidelines” were the most frequently referred-to resources. Much of the advice given in both the Heart Foundation guidance such as the “Healthy Heart” information [16], and the “Diabetes and Healthy Food Choices” pamphlet [17] is consistent with other guidelines and easy to follow. We did not attempt to measure how accurately or consistently these guidelines documents were used. Currently, the most up-to-date Ministry of Health New Zealand guidelines are the 2015 “Eating and Activity Guidelines for New Zealand Adults” [20], specifically written for healthcare practitioners who provide advice on nutrition and physical activity to New Zealand adults, with most of the evidence based on European and North American populations [20]. General practitioners [21] and other healthcare practitioners [10,22] are recognised as having the potential to provide nutrition advice and improve nutrition-related behaviours and risk factors in the context of lifestyle-related long-term conditions; there are few dietetic services available in primary care in New Zealand. However, with the low reported use of guidelines and specific resources, perceived inadequacy of nutrition knowledge amongst participants and an average estimated daily advice frequency of 2.4 times/day in this study, that potential is yet to be realised.

There was general agreement among study participants that a diet high in meat, processed or otherwise, was unhealthy, whereas vegetarian and plant-based diets were perceived as beneficial–although the plant-based diet was not thought easy to follow. For specific foods; dairy was considered the best source of calcium–required for bone strength; meat the best source for protein and iron. Aside from these instances, dairy and high-meat consumption were not seen as healthy. In this research, plant-based diets seemed to be generally supported in principle although there was perhaps a lack of ease with endorsing a diet without dairy and/or meat. Dairy being good for bones/prevents osteoporosis and meat as required for protein and iron appear to be “received beliefs” [23] and as such are open to contest.

In general, although cow’s milk products are rich in calcium, and indeed calcium is one important part of bone strength, there is mixed evidence as to whether dairy foods are helpful or harmful for bone strength or fracture rates [24,25,26]. High dairy consumption carries risks outside of this, for example, milk proteins having been identified as the “dominant causal triggers of type 1 diabetes” [27], and high consumption of cow’s milk is associated with increased acne [28]. Conversely, research has also shown diets high in dairy consumption to be associated with either neutral or slightly decreased risks for type II diabetes [29] and coronary heart disease [30], and decreased risk for colon cancer [31].

High iron intake, especially in the haeme form is related to an increased risk of Coronary Heart Disease [32,33], and red meat (the “best” source) is a class 2A carcinogen. Furthermore, the Adventist Health Study 2 showed that strict vegetarians’ mean iron intake was above minimum recommendations [34], and that average intake amongst vegetarians is similar or greater to omnivores, although omnivores have higher ferritin status. Non-haeme iron absorption varies dependent on stores “from 1 to 23% depending on iron status” and dietary enhancers and inhibitors” [35]. Enhancers include especially Vitamin C/ascorbic acid, and other acids, and inhibitors include dairy, egg yolks, and tannins [36].

Atherosclerosis was widely perceived by study participants (67%) as being potentially reversible by dietary means with agreement (80% agreed or strongly agreed) that a healthy diet was important for managing long-term conditions except for advanced cancer. This reflects the evidence currently on hand that advanced cancer is not known to be amenable to dietary intervention, and that advanced atherosclerotic heart disease has been demonstrated to be reversible [13]. The plant-based diet was perceived to be difficult to follow and commonly perceived barriers to a plant-based diet (from this and other research) include cost, access to foods, difficulty in preparing meals, and cultural attitudes, although the cost may not necessarily increase on a plant-based diet [12]. A survey of 415 Australian adults found that the main barrier to beginning a plant-based diet was lack of information [37], but 79% would be interested in using a plant-based diet to decrease their saturated fat intake, 70% believed it would help them prevent diseases such as cancer and heart disease, 63% thought it would help them control their weight, and 67% thought it would help increase their vitamin and mineral intake. More education for health professionals on various dietary patterns, including specific health benefits of plant-based diets, and how to advise on plant-based diet implementation may be helpful.

A strength of this study was the mixed-method design. This allowed for the collection of general data about the nutrition beliefs held by the study group from the survey, whilst the focus group provided an in-depth analysis of specific discussion points about nutrition. It was important to gain the perspectives of a wide cross-section of health professionals as this gave greater breadth to the study and helped to explore the different sources from which patients may receive nutrition information. This included reasonable numbers of doctors, nurses, and pharmacists, although only one osteopath and no dietitians. Whilst dietitians are a significant source of nutrition education for the community, despite being approached, no dietitians participated in the study.

This study had several limitations. Clearly, the sample size was small. It involved participants in a geographically confined area where a recent trial of a whole food plant-based diet (WFPB) in a primary care setting had been recently performed [12], almost certainly leading to health professionals being more aware of the benefits of a plant-based diet. The results should therefore be considered carefully before extrapolation to a wider New Zealand population. The self-selecting sample may over-represent those who are interested in nutrition; for example, by including those with both greater training and an interest in providing nutrition advice. The use of a questionnaire presents its own problems such as creating potentially strong selection biases, and biases in sampling accurate information within the target population. Some data points were weak with around half not estimating hours of nutrition education, which may be related to difficulty of distant recall. The data did rely on estimates also, rather than more structured formal measurements. It is also acknowledged that perceived adequacy of nutrition training, self-reporting of such, and attempts to influence nutrition behaviour in consultations involving long-term conditions is influenced by not just nutrition knowledge but also confidence with initiating and managing conversations regarding behaviour change [21,38]. This study did not explore whether better-prepared participants would increase the frequency of conversations involving nutrition during consultations. It may be that healthcare workers are aware of their areas of weakness and may be making appropriate referrals to other members of the multi-disciplinary team, further information on what referrals are being made by whom (e.g. do clinicians less knowledgeable in nutrition refer more frequently to colleagues for nutrition advice?) would be helpful.

## 5. Conclusions

Almost half of the practitioners did not feel that they had adequate nutrition knowledge, and advice was in most cases lacking a clear reference to credible, evidence-based information. High-meat and high-processed meat diets were perceived to be unhealthy, and every effort to pursue healthy diets were mostly thought to be important for chronic disease, except in the case of advanced cancer. Plant-based diets were on the whole viewed as beneficial to health, thought to improve quality of life, be satiating, but were not perceived as easy to follow. This study would be better supported by further research, and does have limited external validity, however, reassuringly many findings here were in keeping with previous research by other authors. This supports previous assertions that our health workforce would benefit from more directed nutrition education along with set competencies in order to address common chronic disease. Education could also include education on plant-based dietary options.

## Figures and Tables

**Table 1:** Times per work-day health professionals provide nutritional advice.

Profession	*n* = 41, (%)	Years Post-Graduation (range)	Advice Per Day (range)
All	41 (100)	19 (1–46)	2.4 (0–10)
Doctor	20 (49)	14 (1–43)	2.8 (0–10)
GP	17	16 (2–43)	2.9 (0–10)
Hospital	3	1	2 (0–5)
Nurse	13 (32)	27 (2–45)	2.7 (0–10)
Pharmacist	7 (17)	19 (1–46)	0.8 (0–2)
Osteopath	1 (2)	2	4

*n* = 41 = total number of respondents, respondent percentages presented in brackets for participant professions. Both years post-graduate and advice per day include range of samples in brackets.

**Table 2:** Perceptions of the adequacy of nutrition training for current role *.

Response	*n =* 40 (%)
Strongly agree	1 (3)
Agree	15 (38)
Neither agree nor disagree	7 (18)
Disagree	15 (38)
Strongly disagree	2 (5)

* The question asked was “Do you feel that you have had the appropriate amount of training in nutrition for your current role?”, *n =* 40 as one person did not respond.

**Table 3:** Perceptions of the healthiness of diets containing the following different foods.

*n =* 41 (%)	Healthy		Neither	Unhealthy	
	Very	Slightly		Slightly	Very
High amounts processed meat	0 (0)	0 (0)	3 (7)	5 (12)	33 (80)
3 or more glasses milk/day	0 (0)	3 (7)	14 (34)	20 (49)	4 (10)
1 or more eggs/day	3 (7)	18 (44)	11 (27)	9 (22)	0 (0)
5–8 servings vegetables/day	37 (90)	2 (5)	1 (2)	0 (0)	1 (2)
3 or more servings dairy/day	2 (5)	8 (20)	13 (32)	16 (39)	2 (5)

**Table 4:** Attitudes regarding chronic illness and desirability of eating a healthier diet.

*n =* 41 (%)	Strongly Agree	Agree	Neither	Disagree	Strongly Disagree
Chronic Illness	19 (46)	14 (34)	7 (17)	1 (2)	0 (0)
**Very Advanced**					
Cancer	2 (5)	5 (12)	16 (39)	8 (20)	10 (24)
Heart Disease	11 (27)	12 (29)	12 (29)	4 (10)	2 (5)
Kidney Disease	8 (20)	17 (41)	10 (24)	4 (10)	2 (5)

**Table 5:** Perceptions regarding the following dietary / lifestyle approaches.

	Perceptions of Various Diets n (%)				
	Beneficial		Neither	Harmful	
Type of Diet	Very	Slightly		Slightly	Very
High in meat	0 (0)	1 (2)	7 (17)	23 (56)	10 (24)
Vegetarian	10 (24)	20 (49)	11 (27)	0	0
Vegan	7 (17)	16 (39)	15 (37)	3 (7)	0
Plant-based	12 (29)	13 (32)	13 (32)	3 (7)	0

**Table 6:** Perceptions regarding a plant-based dietary/lifestyle approach.

	Perceptions of a Plant-Based Diet *n* (%)				
As a Dietary Approach (n)	Strongly Agree	Agree	Neither	Disagree	Strongly Disagree
Complicated (40)	1 (3)	17 (43)	10 (25)	12 (30)	0 (0)
Easy (39)	1 (3)	7 (18)	12 (31)	17 (44)	2 (5)
Filling/satiating (39)	4 (10)	15 (38)	14 (36)	5 (13)	1 (3)
Improves life quality (40)	7 (18)	13 (33)	17 (43)	2 (5)	1 (3)
